# *Oldies but Goldies* mtDNA Population Variants and Neurodegenerative Diseases

**DOI:** 10.3389/fnins.2018.00682

**Published:** 2018-10-12

**Authors:** Patrick F. Chinnery, Aurora Gomez-Duran

**Affiliations:** ^1^Department of Clinical Neurosciences, School of Clinical Medicine, University of Cambridge, Cambridge, United Kingdom; ^2^Medical Research Council-Mitochondrial Biology Unit, Cambridge Biomedical Campus, Cambridge, United Kingdom

**Keywords:** mtDNA, haplogroups, PD, LHON, neurodegenerative diseases

## Abstract

mtDNA is transmitted through the maternal line and its sequence variability, which is population specific, is assumed to be phenotypically neutral. However, several studies have shown associations between the variants defining some genetic backgrounds and the susceptibility to several pathogenic phenotypes, including neurodegenerative diseases. Many of these studies have found that some of these variants impact many of these phenotypes, including the ones defining the Caucasian haplogroups H, J, and Uk, while others, such as the ones defining the T haplogroup, have phenotype specific associations. In this review, we will focus on those that have shown a pleiotropic effect in population studies in neurological diseases. We will also explore their bioenergetic and genomic characteristics in order to provide an insight into the role of these variants in disease. Given the importance of mitochondrial population variants in neurodegenerative diseases a deeper analysis of their effects might unravel new mechanisms of disease and help design new strategies for successful treatments.

## Mitochondria And Oxphos

Mitochondria, from ancient Greek, *mito* (thread), and *chondros* (grain), are highly dynamic organelles in continuous communication with the rest of the cell, that mediate several key cellular functions (Wai and Langer, 2016). These include being the primary source of cellular energy, in the form of adenosine triphosphate (ATP), regulating levels of calcium (Grishanin et al., 1996), reactive oxygen species (ROS) (Boveris and Chance, 1973) and apoptosis (Green and Reed, 1998). Mitochondria play a central role in several fundamental metabolic pathways including the tricarboxylic acids cycle (TCA), fatty-acid β-oxidation, and the pyrimidine biosynthesis (Attardi and Schatz, 1988).

Mitochondria generate the vast majority of cellular energy through the oxidative phosphorylation system (OXPHOS), combining respiration with the synthesis of ATP. Cellular respiration is an ordered chain of redox reactions using reducing equivalents [Nicotinamide adenine dinucleotide (NADH) and Flavin adenine dinucleotide (FADH2)] produced from the degradation of carbohydrates to convert oxygen into water. These reactions are carried out by four multi-protein enzymes: the complexes of the electron transport chain (ETC) I, II, III, and IV and two “shuttles”: ubiquinone (coenzyme Q10) and cytochrome c (Saraste, 1999; Smeitink et al., 2000). The energy released in this process is used to pump protons (H^+^) through complex I (4 H^+^), III (4H^+^), and IV (2H^+^), into to the inter-membrane space, generating a positive electrochemical gradient which drives the transport back to the matrix through the complex V or *ATP* synthase. This complex acts as a proton channel that returns the protons to the mitochondrial matrix. The proton flux provides the energy needed to bind adenosine di-phosphate (ADP) and inorganic phosphate into ATP (Mitchell, 1961).

## Mitochondrial Genome

One of the principal features of mitochondria is that they contain their own genetic system. In humans this is a double chain circular molecule, 16.6 Kb long, which codes for 13 proteins of the OXPHOS system, and the 22 tRNAs and 2 rRNAs required for their expression (Taanman, 1999). Seven of the 13 subunits contribute to complex I (ND1, ND2, ND3, ND4, ND4L, ND5, and ND6), one to the complex III (CYB), three to the complex IV (CO1, CO2, and CO3), and two to the complex V (ATP6 and ATP8). mtDNA is composed of 2 chains, heavy (H) and light (L) with different density based on the G/C composition. Most of the genes are coded by the heavy chain including 2 rRNAs, 14 tRNAs and 12 polypeptides), while 8 tRNAs and only one polypeptide (ND6) are encoded by the light chain (Montoya et al., 1982).

mtDNA is polyplasmic, with each mitochondria containing several copies. A somatic cell can contain between hundreds to thousand mtDNA copies depending on the cell type (Robin and Wong, 1988). Usually all of the molecules are identical (homoplasmy). The presence of more than one type or allele of mtDNA in a cell is known as heteroplasmy (DiMauro et al., 1993). The proportion of a heteroplasmic mutation can vary from cell to cell and selective pressures can influence both of these processes (Taylor and Turnbull, 2005; Stewart and Chinnery, 2015; Burr et al., 2018).

**FIGURE 1:**
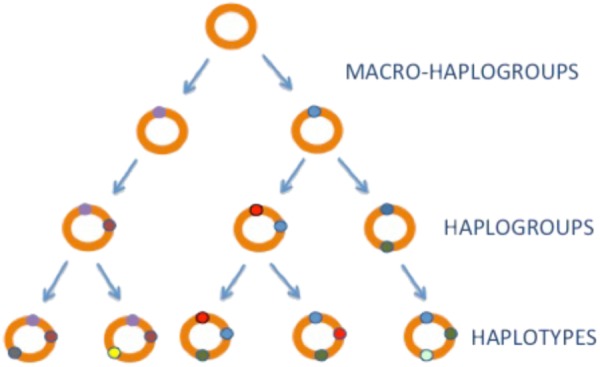
mtDNA inheritance. mtDNA haplogroups. mtDNA is shown as a orange circle, mtDNA variants with little circles in colors.

Mitochondrial DNA is strictly maternally inherited (Giles et al., 1980; Pyle et al., 2015) and has a mutation rate, 5–10 times higher than the nuclear genome (nDNA) (Brown et al., 1995). These factors have led to the accumulation of a wide range of polymorphisms across the mtDNA sequence that are restricted to geographically isolated populations throughout the globe. Given that these genetic variants are inherited from mother to offspring without any recombination, they sequentially accumulate along the radiating maternal lineages (**Figure 1**). This had generated phylogenetically related haplotypes (**Figure 1**). The most common (>1% frequency in the population) are known as mitochondrial haplogroups (Wallace et al., 1999; Wallace, 2015).

## Ancient Polymorphisms Mtdna Haplogroups

**FIGURE 2:**
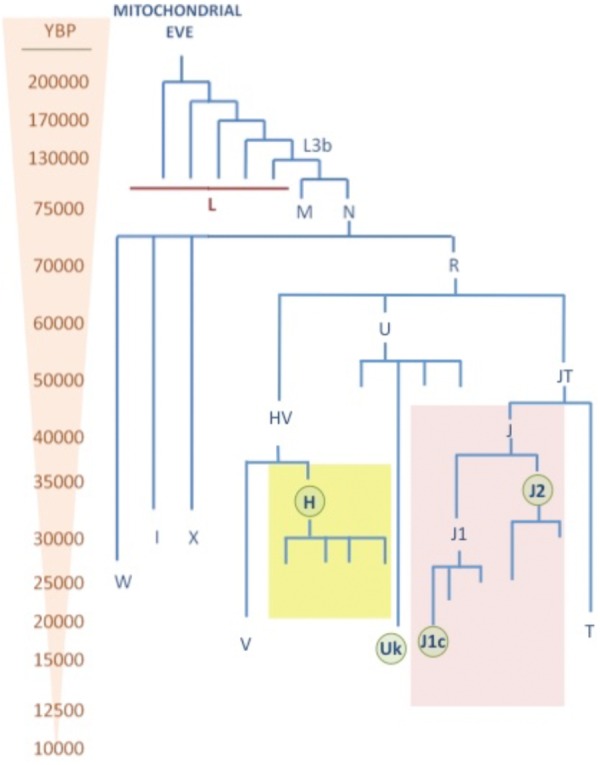
mtDNA phylogenetic tree. This tree shows the age (YBP) of different mtDNA haplogroups. The major Caucasian haplogroups H, U, and J are highlighted in yellow, blue, and red, respectively. Sub-haplogroups which has shown pleiotropic effect in neurodegenerative diseases are highlighted with green circles.

The human mtDNA phylogeny clusters into a three with “unique” ancestor, known as the “Mitochondrial Eve,” rooted in Africa about 150,000 years before present (YBP) (Cann et al., 1987). From this root, four lineages specific for sub-Saharan Africa: L0, L1, L2, and L3 were generated about 100,000 YBP. Then, 60,000 YBP the African haplogroup L3 diverged into the two recent macro-haplogroups M and N, which define populations which left Africa to populate the rest of the world (Soares et al., 2012). During this migration, the haplogroup N was directed to Eurasia and Asia and America, while the M went exclusively to Asia giving place to the haplogroups A, B, C, D, G, and F. In Europe, the haplogroup N 60,000 YBP gave rise to the haplogroup R (Gandini et al., 2016), which is the root of the “European” haplogroups U (60,000 YBP), J (40,000 YBP), T (20,000 YBP), H (30,000–5,000 YBP) (Achilli et al., 2004), and V (15,000 YBP) (Torroni et al., 1996) (**Figure 2**). With the implementation of the next generation sequencing techniques (NGS) there has been a huge increase in the number of mtDNA genomes sequenced and the branches of the tree have diverged into many sub-haplogroups (Brotherton et al., 2013). The current global mtDNA phylogenetic tree contains more than 4,000 different haplogroups (van Oven and Kayser, 2009).

In Europe, 90% of the population belongs to the macro-haplogroups HV, U, and JT (Macaulay et al., 1999; Torroni et al., 2000). The macro-haplogroup HV represents more than 50% of the population, it comprises the haplogroups H and V and HV^∗^. Among them, the haplogroup H an extremely widely distributed and has the highest frequency reaching 45% in Europe, 20% in Turkey and the Caucasus, and around 10% in Gulf countries (Roostalu et al., 2007). It is formed by more than 90 sub-haplogroups (van Oven and Kayser, 2009), where the H1 is the major one having two peaks of high frequency in Scandinavian Peninsula and Southern Iberian Peninsula. The second most frequent haplogroup is the H3, commoner in South of Europe, particularly in France and Spain. The rest of the sub-haplogroups, as for example H2 and H6, are more frequent in the Caucasus and Eastern Europe (Pereira et al., 2005). On the other hand, haplogroup V is found in 4% of the population, principally in European populations, but also present in the north of Africa (Coudray et al., 2009).

The other two macro-haplogroups are the JT and U which account 40% of the European population. Haplogroup U is divided into several sub-haplogroups that make up 20% of the Caucasian population, whereas the subhaplogroups U5 and Uk comprise 9% (Montiel-Sosa et al., 2006). This clade is widely spread from Portugal to India and North of Africa (Achilli et al., 2005). The macro-haplogroup JT includes haplogroups J and T (Ruiz-Pesini et al., 2004). Haplogroup T is divided also in several haplogroups and embody 8% of the population (SanGiovanni et al., 2009). Haplogroup J is found in 9% of Europeans, and is divided into 2 principal sub-haplogroups J1c and J2 (Carelli et al., 2006).

## Mtdna Polymorphisms And Neurological Diseases

Over the last 20 years, many studies have found associations between inherited mtDNA population variants and neurological diseases. Since the first association of Leber Hereditary Optic Neuropathy (LHON) with the mitochondrial haplogroup J in the late 90s (Torroni et al., 1996; Carelli et al., 1997; Hofmann et al., 1997; Lamminen et al., 1997), many other mitochondrial disorders along with classic neurodegenerative diseases like Parkinson (PD), Alzheimer (AD), Multiple Sclerosis (MS), and Amyotrophic Lateral Sclerosis (ALS).

### Mitochondrial Diseases

Mitochondrial diseases are a large group of heterogeneous disorders caused by mutations in the mtDNA and the nuclear DNA (nDNA). Although, their phenotypes are hugely variable, some of them overlap with clinical phenotypes observed in neurodegenerative diseases (Betts et al., 2004; Swerdlow, 2009). For example, neurodegeneration in the cerebellar purkinje layer and cortical neurons in the occipital and parietal lobes has been observed in patients with Mitochondrial encephalomyopathy, lactic acidosis, and stroke-like episodes (MELAS) (Betts et al., 2004). Leber Hereditary Optic Neuropathy (LHON) disorder is characterized by neurodegeneration of the retinal ganglion cell (RGC) layer and optic nerve (Yu-Wai-Man et al., 2011). In addition, a small sub-set of LHON patients develop similar signs to multiple sclerosis, in a disease known as Harding’s disease (Pfeffer et al., 2013; Bargiela and Chinnery, 2017) and signs of parkinsonism have also been observed in LHON pedigrees (Simon et al., 1999; Vital et al., 2015).

#### Leber Hereditary Optic Neuropathy

LHON is a mitochondrial neurodegenerative disorder characterized by RGC dysfunction and rapid visual loss. In Caucasian population, approximately 90% of LHON cases are caused by a mutation in the *MT-ND* genes subunits encoding for the mitochondrial complex I; m.11778G > A:*MT-ND4* (60%), m.3460G > A:*MT-ND1* (15%) and m.14484T > C:*MT-ND6* (15%). The remaining 10% of the LHON cases harbor rarer mutations (Achilli et al., 2012; Maresca et al., 2014; Caporali et al., 2018). Although the primary genetic cause of LHON is known, the presence of a mtDNA mutation is not enough on its own to cause the blindness. Many factors such as gender, with higher penetrance in males than females, genetic factors including the mitochondrial haplogroups, and other environmental factors have been related with the clinical penetrance of the disease (Kirkman et al., 2009; Caporali et al., 2017).

The role of mtDNA population variants in LHON has been widely studied. Initial studies described the variants m.4216C > T:*MT-ND1* and m.13708G > A:*MT-ND5*, m.15257G > A:*MT-CYB*, m.15812G > A:*MT-CYB* defining J and J2 haplogroup, respectively, as “secondary” LHON mutations (Johns and Berman, 1991; Johns and Neufeld, 1991; Huoponen et al., 1993; Oostra et al., 1994; Brown et al., 1995; Harding et al., 1995; Howell et al., 1995; Lodi et al., 2000), before the association was described with haplogroup J (Carelli et al., 1997; Hofmann et al., 1997; Lamminen et al., 1997; Torroni et al., 1997). Studies with bigger cohorts in Italian population (86 cases) narrowed down the associations to specific LHON mutations, where the m.11778G > A:*MT-ND4* mutation was over-represented in subhaplogroups J1c and J2b defined by variants in the *MT-CYB*; and m.14484T > C: *MT-ND6* was over-represented in haplogroup J1 (Carelli et al., 2006). The role of the *MT-CYB* variants was also confirmed in the most comprehensive study carried out to date. In a cohort counting with 3,613 individuals from 159 European LHON families, Hudson et al. confirmed mutation specific sub-haplogroup associations (Hudson et al., 2007). Individuals carrying the mutation m.3460G > A: *MT-ND1* had an increase of risk in the haplogroup Uk [OR = 2.37, *p* = 0.002, CI 95% (1.36–4.13)]. Individuals carrying m.11778G > A: *MT-ND4* had an increased risk in the J haplogroup [OR = 1.31, *p* = 0.02, CI 95% (1.03–1.65)], and a reduced risk on a haplogroup H background [OR = 0.79, *p* = 0.04, CI 95% (0.63–0.98)] (Hudson et al., 2007).

Recent studies have shown the combinations of polymorphisms may lead to a reduced OXPHOS efficiency and be sufficient to trigger LHON (Achilli et al., 2004; Caporali et al., 2018). This is in keeping with our recent findings, where we showed that some mtDNA sequences appear to influence the probability of acquiring new pathological mutations in a population specific manner (Wei et al., 2017).

#### Other Mitochondrial Diseases

Independently from LHON studies, other mitochondrial disorders such as MELAS, neuropathy, ataxia, retinitis pigmentosa (NARP)/Maternally inherited Leigh’s syndrome (MILS), and age-related hearing loss (ARHL) have been associated with certain mtDNA haplogroups, but these findings remain controversial.

In the case of MELAS, a study in 142 unrelated French families carrying the m.3243A > G mutation observed a statistically significant under-representation of the mutation in haplogroup J patients [OR = 0.26, *p* = 0.01, CI 95% (0.08–0.83)] (Pierron et al., 2008). Analysis of the same mutation in smaller sample from Spanish population did not find any association (Torroni et al., 2003).

Mitochondrial haplogroups do not appear to play a role in non-syndromic deafness caused by the m.1555G > A (Torroni et al., 1999), although an association with haplogroup H3 was described in one small cohort of Spanish mutation carriers (Achilli et al., 2004). The analysis of 912 ARHL patients found that haplogroup U was significantly associated with moderate to severe phenotype (OR 3.02; CI 95%: 1.30–6.99); and in patients aged from 50 to 59 years sub-haplogroup Uk was associated with severe ARHL only (OR 3.02; CI 95%: 1.30–6.99) (Manwaring et al., 2007). Although it is difficult to draw firm conclusions from these small single studies, in line with these findings, a study in transmitochondrial cell lines carrying the mutation m.8993T > G: *MT-ATP6* responsible NARP/MILS syndrome showed an significant increase severity of the OXPHOS defect in cell lines from the haplogroup U5b compared those belonging to haplogroup H (D’Aurelio et al., 2010).

### Parkinson Disease

The association of mtDNA polymorphisms and Parkinson’s disease pathogenesis has been controversial, although recent large studies have validated the original findings in independent cohorts. Many studies have shown association between PD and particular haplogroups (Moilanen et al., 2001; Ross et al., 2003; van der Walt et al., 2003; Otaegui et al., 2004a; Ghezzi et al., 2005; Huerta et al., 2005; Pyle et al., 2005; Gaweda-Walerych et al., 2008; Khusnutdinova et al., 2008; Latsoudis et al., 2008; Georgiou et al., 2017), while others could not directly replicate these findings (Simon et al., 2010; Fachal et al., 2015). Most of the studies showed population specific associations. Indeed, an early work in Finns found a reduced risk for PD in the supercluster HVKU compared to the supercluster JTIWX which was exclusively associated with the Uk cluster (Autere et al., 2004). Studies in a Polish population of 241 PD patients and 277 control subjects, didn’t find differences between the haplogroups, however, after stratification by gender, they found that haplogroup J [OR = 0.19, *p* = 0.0014, CI 95% (0.069–0.53)] was associated with a lower PD risk in males (Gaweda-Walerych et al., 2008). Another study in an Italian population comprising of 620 idiopathic PD patients and about 2000 controls found a role of haplogroup Uk in decreasing the penetrance of PD [OR = 0.54, *p* = 0.048, CI 95% (0.35–0.83)] (Ghezzi et al., 2005), while in an study on a Spanish cohort of 271 PD patients and 230 healthy controls significant association was found for the polymorphism defining the haplogroup H5 mt.4336 T>C:*tRNA*^Gln^ with a significantly increased frequency in PD compared to controls [OR = 4.45, *p* = 0.011, CI 95% (1.23–15.96)] but only in females (Huerta et al., 2005). Other study in a Tatar Russian population of 183 unrelated PD patients and 157 controls found that polymorphisms associated with haplogroup H mtDNAs increased PD risk (OR = 2.58, *p* = 0.0001), whereas those associated with haplogroup Uk were protective (OR = 0.38, *p* = 0.003) (Khusnutdinova et al., 2008). Conversely, the Irish study that included 90 Irish PD patients and 129 Irish controls, reported that haplogroups J and T increased PD risk (Ross et al., 2003).

The variability of results could have been affected by population stratification issues, small sample sizes and variations in the statistical approach used. However, three recent meta-analysis have shown evidence of the existence of both protective and risk haplogroup alleles associated with PD. The first study counted with 3,074 PD cases and 5,659 ethnically matched controls followed by meta-analysis of 6,140 PD cases and 13,280 controls. They found that two variants, m.2158T > C:*MT-RNR2* and m.11251A > G:*MT-ND4*, which are phylogenetically linked and define the mitochondrial superhaplogroup JT and sub-haplogroup J1b, respectively, were associated with a reduced risk of PD [OR = 0.87, CI 95% (0.77–0.97)] (Hudson et al., 2013). In line with this finding, a second study from the same group, including 2,197 patients compared with three independent control groups genotyped on the same platforms (C1 = 2997, C2 = 2897, and C3 = 5841 control samples), also showed a protective effect for the variant m.10398A > G:*MT-ND3*, homoplastic (recurrent in several branches of the mtDNA phylogenetic tree) on haplogroups J and Uk, which was previously associated with reduced risk to PD (Ghezzi et al., 2005; Huerta et al., 2005; Hudson et al., 2014). Finally, a meta-analysis from 13 different studies combining *n* = 9243 patients and n = 17,999 controls found that individuals mtDNA haplogroup Uk [OR = 0.839, *p* = 0.004, CI 95% (0.744–0.945)], haplogroup T [OR = 0.857, *p* = 0.014, CI 95% (0.757–0.969)] and haplogroup J [OR = 0.876, *p* = 0.011, CI 95% (0.79–0.971)] had a significantly reduced risk of developing PD, while the macro-haplogroup HV act as a increased risk factor [OR = 1.091, *p* = 0.038, CI 95% (1.005–1.184)] (Marom et al., 2017).

### Alzheimer Disease

The implication of mtDNA variants in AD remains contentious. Several studies have reported haplogroup associations (Chagnon et al., 1999; Carrieri et al., 2001; Fesahat et al., 2007; Santoro et al., 2010; Maruszak et al., 2011; Coskun et al., 2012; Ridge et al., 2012) but the results have been inconsistent, and others found no evidence of an association (Chinnery et al., 2000; Hudson et al., 2012; Fachal et al., 2015; Pyle et al., 2015) (for extensive review of the data see Ridge and Kauwe, 2018). The largest study for mtDNA carried with 3,250 AD patients and 1,221 controls found no significant association between mtDNA variants and AD (Hudson et al., 2012). However, a more recent meta-analysis involving several studies did report an increased risk of AD in people belonging to haplogroup H [OR = 1.283, *p* = 0.016, 95% CI (1.047–1.574)] (Marom et al., 2017), but larger studies are warranted to validate these findings which only just reach conventional statistical significance.

In addition to the genetic analysis, a study from the Alzheimer’s Disease Neuroimaging Initiative (ADNI) database including 175 controls and 154 AD patients found that individuals from U5b1 and Uk1a1b haplogroups had grater rates of temporal pole atrophy, an endophenotype of AD risk (Ridge et al., 2013).

### Multiple Sclerosis

Following the trend of the previous disorders, the data on associations between mitochondrial haplogroups and MS is contradictory. While some small studies have seen an increase in the risk associated with the haplogroup Uk in different populations (Kalman et al., 1999; Hassani-Kumleh et al., 2006; Vyshkina et al., 2008), others have shown a consistent association with haplogroup J (Kalman et al., 1999; Houshmand et al., 2005; Mihailova et al., 2007), and others failed to find any association in United Kingdom (Ban et al., 2008) and Basque population (Otaegui et al., 2004b).

Larger studies have, however, found consistent associations, mainly with the macro-haplogroup JT as a risk factor in European populations. The first meta-analysis studying more than 2500 MS samples and the same number of controls found that the variant m.13708G > A: *MT-ND5*, defining the haplogroup J, was significantly associated with an increased risk to MS [OR = 1.71, *P* = 0.0002, CI 95% (1.29–2.27)] (Yu et al., 2008). Similarly, another recent meta-analysis involving 7,391 and 14,568 controls confirmed the association between haplogroup J (OR = 1.11, *p* = 0.03, CI 95% (1.01–1.22)] and haplogroup T carriers [OR = 1.17, *p* = 0.002, CI 95% (1.06–1.29)]. In the same study, only 3 populations (Italy, United Kingdom, and Germany) showed an association between primary progressive (PP) PPMS and haplogroup J [OR = 1.49, *p* = 0.009, CI 95% (1.10–2.01)], but a smaller cohort with 3,720 cases and 879 controls from US did not show the same association (Tranah et al., 2015). These findings could be due to differences in the frequency of sub-haplogroups in the populations studied (Andalib et al., 2015).

### Amyotrophic Lateral Sclerosis

The larger study carried out in ALS in a cohort of 700 patients and 462 controls from two European populations did not find any association between mtDNA haplogroups and ALS (Ingram et al., 2012). Similar results were previously obtained in a cohort from UK population of 504 ALS patients and 493 controls (Chinnery et al., 2007). Conversely, in a cohort from Italian population with 222 patients with sporadic ALS (sALS) and 151 matched controls, the haplogroup I demonstrated to be associated a higher risk of ALS when compared to the common haplogroup, H [OR: 0.08, *p* < 0.01, CI 95% (0.04–0.4)] (Mancuso et al., 2004). Overall, studies of mtDNA haplogroups in ALS remain much smaller than other neurodegenerative diseases.

## From Epidemiology To Bioenergetics

**FIGURE 3:**
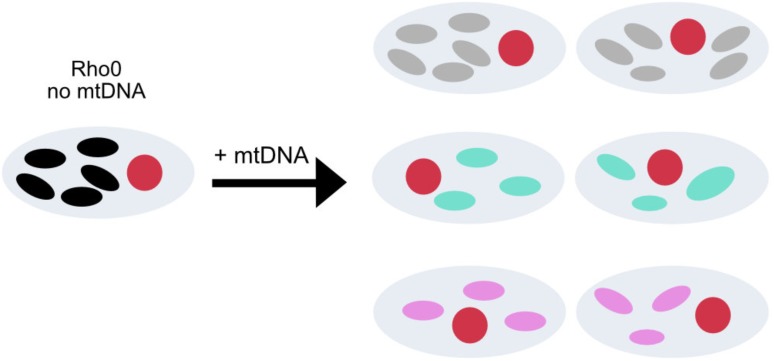
Transmitochondrial cybrid model. Construction of cybrid cell lines containing the same nuclear DNA, but mtDNA from healthy individuals with different mtDNA haplogroups. The oval circles inside the cell with colors white, gray, blue, pink and black represent no mtDNA and mtDNA from with different mtDNA, respectively.

Despite earlier contention, there is now clear evidence that mtDNA variants within haplogroups are associated with specific neurodegenerative disorders, with the most consistent evidence for Parkinson disease and LHON. This raises the question: how do these genotypes mediate their pleiotropic effects? Functional studies are technically challenging because the biochemical effect of common polymorphisms is likely to be subtle. In addition, given that many of the associated variants are found on genomes containing other polymorphisms, several linked alleles may be interacting. Unfortunately, the double mitochondrial membrane precludes site directed mutagenesis at present, so functional studies are limited to mtDNA harvested from human cells. The generation of transmitochondrial cybrids present one approach to address some of these issues (Chomyn et al., 1994). A cybrid gives the opportunity of studying the effect of a determined mtDNA in a fixed nuclear background under the same ambient conditions. Briefly, a rho0 cell line is generated by removing mtDNA (King and Attardi, 1996; Miller et al., 1996), can be fused with enucleated fibroblasts (King and Attardi, 1989) or with platelets containing the mtDNA of interest (Chomyn et al., 1994; **Figure 3**).

Cybrid technology has been widely used for the study of phenotypical effect of inherited pathogenic mutations in the mtDNA (Hayashi et al., 1991; Trounce et al., 1994; Vergani et al., 1995; Brown et al., 2001; D’Aurelio et al., 2001), but the model itself raises several concerns, including aneuploidy and an unstable nuclear background in a cancer derived cell line, the use of mutagenic agents for the generation of rho0, and biochemical properties of the original tumor (for further information see Iglesias et al., 2012). However, given the benefits shown when using this approach to elucidate disease mechanisms for mitochondrial diseases, the technique has been extensively applied for the study of the mtDNA population variants to look for subtler biochemical effects of defined genetic variants.

Cybrids from haplogroup H contain higher mtDNA levels and mtDNA encoded mRNA levels, growing faster, have a higher membrane potential, and consume more oxygen per ETC unit than cybrids from haplogroup Uk individuals (Gómez-Durán et al., 2010). Similar differences were found when characterizing cybrids from haplogroup H and T in HEK293 cells background, where H cybrids showed higher respiration capacity per molecule of mtDNA, compared to HEK293 cybrids, but lower growth rate (Mueller et al., 2012). Contrarily, in another study, Caucasian haplogroups H and T showed no differences in the mitochondrial membrane potential and the oxygen consumption per cell. Other analysis didn’t find differences between the “artic” (A, C, and D) and the “tropical” haplogroups (L1, L2, and L3) or the European haplogroups H and T (Amo and Brand, 2007; Amo et al., 2008).

Haplogroup J has been widely linked to several diseases, and therefore deeply characterized using different nuclear genetic backgrounds. Similarly, to the epidemiological studies, initial work with a small number of samples showed no functional differences between the haplogroup H, J, and T (Carelli et al., 2002). However, larger studies showed that haplogroup J cell lines have slower rate of assembly of the mitochondrial complexes (Pello et al., 2008) on a nuclear 143B background. In studies in Wal-2A cell line haplogroup J1b revealed higher mtDNA levels and TFAM binding than a cell lines from haplogroup H (Suissa et al., 2009). However, using ARPE-19 cells, the same group did not find differences in the mtDNA levels between haplogroups H and J, but found lower levels in ROS production and ATP levels in haplogroup J (Kenney et al., 2013). Similarly, J cybrids on a 143B background showed less lower ATP and ROS production than haplogroup H cybrids (Bellizzi et al., 2012). This contrasts with another study of 9 cybrid lines from the haplogroup J, which were compared to 5 from the haplogroup H, and did not find any difference in manganese superoxide dismutase (MnSOD) expression, a marker of reactive oxidative species (ROS) production. This study did, however, confirmed the previous findings of lower oxygen consumption and low total ATP levels in the haplogroup J cell lines (Gómez-Durán et al., 2012).

Besides the discrepancies among the studies, it seems that cell lines carrying the mitochondrial haplogroup J have less OXPHOS capacity and ATP levels than those from the haplogroup H. In keeping with this, *in vivo* studies of individuals showed that individuals from the J haplogroup were shown to have lower maximal oxygen uptake (VO2max) (Marcuello et al., 2009) and haplogroup H higher (Martinez-Redondo et al., 2010) in an Spanish cohort.

The variation observed in all studies between the haplogroups carried *in vitro* could be due to 2 factors; the effect in the nuclear background uses and/or to the differences between the sub-haplogroups of each haplogroup (Chen et al., 2012). Indeed, while in our studies we studied cells carrying mtDNA from the subhaplogroups of the J; J1b1 (1), J1c (4), J2 (4), H1 (3), H5 (1), and H1 (3) (Gómez-Durán et al., 2012), Suissa et al. and the Kenney et al. included J1 (1), J1b2 (1), H1 (1), H6 (2) and H^∗^(2) and J1c (2), J1d1a (1), and H subhaplogroups that were not stated, respectively, (Suissa et al., 2009; Kenney et al., 2013). Thus, the observed inconsistency could be due to the variants in the younger branches of the phylogenetic tree that define the sub-haplogroups (Hudson et al., 2014). Altogether, this could also affect nDNA-mtDNA retrograde response signaling in a haplogroup dependent manner.

## Mitochondrial Signaling In Mtdna Population Variants

Mitochondrial nuclear crosstalk was first described in yeast after the depletion of mtDNA which induce the expression of transcription factors (Butow and Avadhani, 2004; Picard et al., 2013). Mitochondrial dysfunction also induces retrograde responses (Chae et al., 2013). The biochemical disruption alter many downstream including immune signaling (Picard et al., 2015), mTORC1 (Khan et al., 2017), AMP-activated protein kinase (AMPK) (Zheng et al., 2016) and the transcription factors ATF4 (Quiros et al., 2017) and/or ATF5 (Fiorese et al., 2016; Suomalainen and Battersby, 2017).

mtDNA population variants also have been shown to affect a variety of signaling pathways. Cybrids in 143B osteosarcoma background from haplogroup J showed higher expression levels of IL-1β and TNFR2 than the ones from haplogroup H (Bellizzi et al., 2006). In the same cell lines J haplogroup showed an increase expression of the methionine adenosyltransferase 1A (*MAT1A*) gene, and therefore an increase in the global methylation compared to the cells from the H haplogroup (Bellizzi et al., 2012). In ARPE-19, cybrids cell lines from the haplogroup J have reduced Complement factor H (*CFH*), Complement component 3 (*C3)* and expression levels than those from haplogroup H (Kenney et al., 2013) and increase in apoptotic genes like *RAR* (Kenney et al., 2014). Another study in 143B cells showed a significantly increased expression of BBC3 in H cybrids compared with J cybrids (Fernández-Moreno et al., 2017). We have seen an increase in the expression of phosphofructokinase in the cell lines from the haplogroup J and Uk when compared to cells from the haplogroup H (Gómez-Durán et al., 2010, 2012).

In addition, a recent study has shown that the variant defining haplogroup J m.13708G > A: *MT-ND5* is 2 bases upstream of a methylation RNA site at the gene *MT-ND5* (13710) which severely reduces the capacity of *MT-ND5* to be methylated and its predicted to have phenotypical implications (Safra et al., 2017).

## Future Perspectives

In summary, the large number of studies on mtDNA population variants over the last 25 years have clearly shown that mtDNA polymorphisms defining the mitochondrial haplogroups are certainly not phenotypically neutral as previously assumed. Extensive work has shown their role in evolution, disease, bioenergetics and cell signaling. However, understanding how the same variant could be advantageous or detrimental in different contexts, and their effect in mtDNA-nDNA communication needs to be further understood. An important caveat is that many of the published studies could be false positive results, in part related to multiple significance testing. To date, there has been limited or no attempt to independently replicate the original findings. Confirmatory replication has been possible in some instances, most notably Parkinson’s disease (Hudson et al., 2013; Marom et al., 2017), but there is a need for large well-powered discovery and replication studies to validate many of the findings discussed above, accounting for multiple significance testing. Another challenge is the phylogenetically related nature of the mtDNA which makes difficult to determine how the dependence between the variants influences the associations. The recent increase of available data in NGS for whole genome sequence studies (including mtDNA) in combination with accessible public data from other “omic” technologies such as proteomics, metabolomics and transcriptomics will definitely make a difference helping understanding which (“goldies”) variants are responsible for the associations. Given the complexity of these pathologies, a combination of efforts between multidisciplinary teams combining genomics studies on patient samples in combination with wet lab studies on cell biology and biochemistry will pave the way to elucidate the mechanisms of their role in cellular metabolism and dysfunction. This will lead to enhanced understanding of mtDNA heritability in neurodegenerative diseases and open new avenues for effective treatments.

## Author Contributions

AG-D designed and wrote the manuscript together with PFC.

## Conflict of Interest Statement

The authors declare that the research was conducted in the absence of any commercial or financial relationships that could be construed as a potential conflict of interest.
